# Detection of Viable *Mycobacterium ulcerans* in Clinical Samples by a Novel Combined 16S rRNA Reverse Transcriptase/IS*2404* Real-Time qPCR Assay

**DOI:** 10.1371/journal.pntd.0001756

**Published:** 2012-08-28

**Authors:** Marcus Beissner, Dominik Symank, Richard Odame Phillips, Yaw Ampem Amoako, Nana-Yaa Awua-Boateng, Fred Stephen Sarfo, Moritz Jansson, Kristina Lydia Huber, Karl-Heinz Herbinger, Florian Battke, Thomas Löscher, Ohene Adjei, Gisela Bretzel

**Affiliations:** 1 Department of Infectious Diseases and Tropical Medicine (DITM), University Hospital, Ludwig-Maximilians University, Munich, Germany; 2 Komfo Anokye Teaching Hospital (KATH), Kwame Nkrumah University of Science and Technology (KNUST), Kumasi, Ghana; 3 Kumasi Centre for Collaborative Research (KCCR), Kwame Nkrumah University of Science and Technology (KNUST), Kumasi, Ghana; 4 Dr. Battke SCIENTIA GmbH, Taufkirchen, Germany; University of Tennessee, United States of America

## Introduction

Buruli ulcer disease (BUD) caused by *Mycobacterium ulcerans* involves the skin and soft tissue. If left untreated, extensive destruction of tissue followed by scarring and contractures may lead to severe functional limitations. Following the introduction of standardized antimycobacterial chemotherapy with rifampicin and streptomycin, recurrence rates of less than 2% were reported. However, treatment failures occur and a variety of secondary lesions necessitating customized clinical management strategies have been reported. True recurrences by definition occur more than three months after completion of antibiotic treatment, are characterised by the presence of viable bacilli, and require a second course of antibiotics. “Non-healers” may harbour viable, possibly drug-resistant *M. ulcerans* strains and may benefit from surgical intervention. Early-onset immune-mediated paradoxical reactions emerging during or shortly after treatment do not contain viable bacilli and may heal under conventional wound care and/or minor surgery; late-onset secondary lesions presumably attributable to secondary infection foci may clear spontaneously through enhanced immune responses primed by initial treatment. None of the current diagnostic techniques is applicable to rapidly address the pivotal question of the presence of viable bacilli in non-healers and patients with secondary BUD lesions, and optimal time points for collection of follow-up samples have not yet been investigated. Therefore, to date treatment monitoring is mainly based on clinical observation [1]–[5]. Reverse transcriptase assays targeting 16S rRNA and mRNA were successfully applied for the rapid detection of viable mycobacteria in clinical samples from patients with tuberculosis and leprosy [6], [7]. To employ this technique for classification of BUD lesions and monitoring of treatment success we developed a *M. ulcerans*–specific RNA-based viability assay combining a 16S rRNA reverse transcriptase real-time PCR (RT-qPCR) to determine bacterial viability with an IS*2404* quantitative real-time PCR (qPCR) for increased specificity and simultaneous quantification of bacilli.

## Development and Validation

### Ethical Approval

The study was approved by the Committee of Human Research Publication and Ethics, School of Medical Sciences, Kwame Nkrumah University of Science and Technology, Kumasi, Ghana (CHRPE/28/09). Written informed consent was obtained from all study participants, or their legal representatives.

### Bacterial Strains, DNA Extracts, and Clinical Samples

Technical validation of the assay was performed with 29 *M. ulcerans* strains originating from Cameroon [8] and Ghana (Table 1), as well as DNA extracts from 18 closely related human pathogenic mycobacterial species and five bacterial species frequently colonizing human skin (Table 2).

**Table 1:** *M. ulcerans* cultures subjected to the 16S rRNA RT/IS*2404* qPCR assay.

*M. ulcerans* Strain	Source	Origin^a^	16S rRNA RT-qPCR^b^	IS*2404* qPCR^c^	IS*2404* qPCR – Wipeout Control^d^
K4s-C1	DITM	Human isolate – Kamerun	Positive	Positive	Negative
K4s-C2	DITM	Human isolate – Kamerun	Positive	Positive	Negative
K4s-C3	DITM	Human isolate – Kamerun	Positive	Positive	Negative
K5d-C1	DITM	Human isolate – Kamerun	Positive	Positive	Negative
K5d-C2	DITM	Human isolate – Kamerun	Positive	Positive	Negative
K5d-C1	DITM	Human isolate – Kamerun	Positive	Positive	Negative
K5d-C2	DITM	Human isolate – Kamerun	Positive	Positive	Negative
K5d-C3	DITM	Human isolate – Kamerun	Positive	Positive	Negative
K5d-C4	DITM	Human isolate – Kamerun	Positive	Positive	Negative
K5s-C1	DITM	Human isolate – Kamerun	Positive	Positive	Negative
K5s-C2	DITM	Human isolate – Kamerun	Positive	Positive	Negative
K5s-C3	DITM	Human isolate – Kamerun	Positive	Positive	Negative
K5s-C4	DITM	Human isolate – Kamerun	Positive	Positive	Negative
K5s-C5	DITM	Human isolate – Kamerun	Positive	Positive	Negative
K7b-C1	DITM	Human isolate – Kamerun	Positive	Positive	Negative
K7b-C2	DITM	Human isolate – Kamerun	Positive	Positive	Negative
K7b-C3	DITM	Human isolate – Kamerun	Positive	Positive	Negative
K7b-C4	DITM	Human isolate – Kamerun	Positive	Positive	Negative
K7s-C1	DITM	Human isolate – Kamerun	Positive	Positive	Negative
K7s-C2	DITM	Human isolate – Kamerun	Positive	Positive	Negative
K12S-C1	DITM	Human isolate – Kamerun	Positive	Positive	Negative
941328-C1	DITM	Human isolate – Ghana	Positive	Positive	Negative
07-C1	DITM	Human isolate – Ghana	Positive	Positive	Negative
DS1-C1	DITM	Human isolate – Ghana	Positive	Positive	Negative
97680-C1	DITM	Human isolate – Ghana	Positive	Positive	Negative
G.A.P.001-C1	KCCR	Human isolate – Ghana	Positive	Positive	Negative
G.A.P.033-C1	KCCR	Human isolate – Ghana	Positive	Positive	Negative
G.A.P.071-C1	KCCR	Human isolate – Ghana	Positive	Positive	Negative
G.A.P.078-C1	KCCR	Human isolate – Ghana	Positive	Positive	Negative

Table 1 shows 29 *M. ulcerans* cultures that were available at the Department of Infectious Diseases and Tropical Medicine (DITM) and the Kumasi Centre for Collaborative Research (KCCR) for development and technical validation of the 16S rRNA RT/IS*2404* qPCR viability assay and the corresponding test results. Sequence analysis of 16S rRNA genes from the listed strains revealed 100% nucleotide concordance of the corresponding genomic regions amplified by the 16S rRNA RT-qPCR; no SNPs or mutations were detected, suggesting a high selectivity of the assay. Sequencing primers are described in Table 3 [11].

a *M. ulcerans* cultures were available from previous studies from Kamerun (*n* = 21) and Ghana (*n* = 4) at DITM [8] or were available at KCCR (*n* = 4) from the present study. All strains were of human origin (BUD patients) and confirmed by conventional IS*2404* PCR and sequencing of *rpoB*- and *rpsL*-genes that revealed the *M. ulcerans* Agy99 wild-type sequences (GenBank accession no. CP000325.1) [11], [12].

b Results of the 16S rRNA RT-qPCR of mycobacterial RNA extracts.

c Results of the IS*2404* qPCR of mycobacterial DNA extracts.

d Results of the IS*2404* qPCR of genomic DNA (gDNA) wipeout controls (see Protocols S2 and S3); a positive result indicates gDNA contamination of RNA extracts following DNAse digestions, and a negative result indicates RNA extracts free of gDNA.

**Table 2:** Specificity of 16S rRNA and IS*2404* qPCR assays.

Bacterial Species	Source^a^	Origin^b^	16S rRNA^d^	IS*2404* e
*M. abscessus*	NRZ	Human isolate^p^	−	−
*M. africanum*	NRZ	Human isolate^p^	−	−
*M. avium*	NRZ	Human isolate^p^	−	−
*M. bovis*	NRZ	Cattle isolate^p^	−	−
*M. chelonae*	NRZ	Human isolate^p^	−	−
*M. fortuitum*	NRZ	Human isolate^c^	−	−
*M. gordonae*	NRZ	Human isolate^c^	−	−
*M. gordonae*	DITM	Human isolate^c^	−	−
*M. kansasii*	NRZ	Human isolate^p^	−	−
*M. leprae*	DITM	Human isolate^p^	−	−
*M. malmoense*	NRZ	Human isolate^c^	−	−
*M. marinum*	NRZ	Human isolate^p^	+	−
*M. microti*	NRZ	Mouse isolate^p^	−	−
*M. scrofulaceum*	NRZ	Human isolate^p^	−	−
*M. smegmatis*	NRZ	Human isolate^p^	−	−
*M. szulgai*	NRZ	Human isolate^p^	−	−
*M. tuberculosis*	NRZ	Human isolate^p^	−	−
*M. ulcerans*	DITM	Human isolate^p^	+	+
*M. xenopi*	NRZ	Human isolate^c^	−	−
*E. coli*	MVP	Human isolate^c^	−	−
*P. acnes*	MVP	Human isolate^p^	−	−
*Staph. aureus*	MVP	Human isolate^c^	−	−
*Staph. epidermidis*	MVP	Human isolate^c^	−	−
*Str. pyogenes*	MVP	Human isolate^p^	−	−

Table 2 shows DNA extracts from closely related mycobacterial species and bacteria potentially contaminating the human skin subjected to the combined 16S rRNA RT/IS*2404* qPCR viability assay and the corresponding test results. Mycobacterial species were selected according to their respective genetic contiguousness to *M. ulcerans* Agy99 (GenBank accession no. CP000325.1) within the 16S rRNA gene sequences as determined by BLASTN analysis (GenBank, NCBI) [13]. *M.*, *Mycobacterium*; *E.*, *Escherichia*; *P., Propionibacterium*; *Staph*., *Staphylococcus*; *Str*., *Streptococcus*. While in-silico analysis revealed that the combined 16S rRNA RT/IS*2404* assay will also amplify mycolactone-producing mycobacteria (MPM) other than *M. ulcerans* (e.g., *M. pseudoshottsii*, *M. liflandii*, and the environmental *M. marinum* [GenBank accession No. NR_042988.1, AY500838.1, and AF456241.1, respectively]), these MPM species were not included in specificity testing.

a DNA extracts that were not available at the DITM were provided by the National Reference Center (NRZ) for Mycobacteria, Borstel, Germany, and the Max von Pettenkofer-Institute (MVP), Ludwig-Maximilians University, Munich, Germany.

b The respective primary patient isolates were considered as ^p^pathogenic bacteria or as ^c^commensals/contaminants of clinical samples.

d Results of the 16S rRNA RT-qPCR of DNA extracts; “+” indicates a positive and “–” a negative test result.

e Results of the IS*2404* qPCR of DNA extracts; “+” indicates a positive and “–” a negative test result.

**Figure 1:**
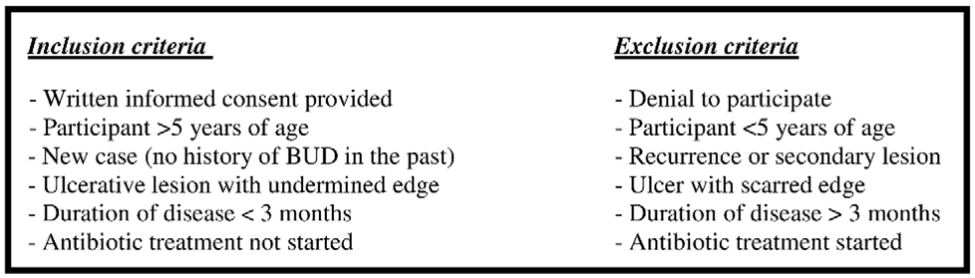
Enrolment criteria for the pre-treatment study population. Figure 1 describes enrolment criteria for clinically suspected BUD patients presenting at Agogo Presbyterian Hospital (*n* = 14) and Tepa Governmental Hospital (*n* = 10), Ghana, respectively. None of the eligible study participants was excluded.

**Figure 2:**
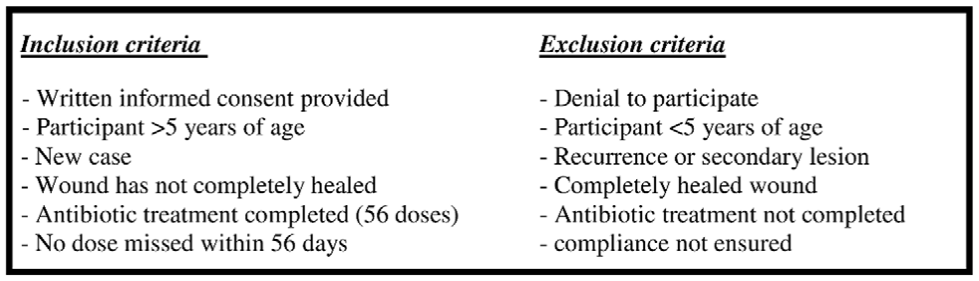
Enrolment criteria for the post-treatment study population. Figure 2 describes enrolment criteria for IS*2404* PCR confirmed BUD patients with incomplete wound healing (collection of swab samples feasible) who presented at Agogo Presbyterian Hospital, Ghana (*n* = 7), following completion of 56 doses of rifampicin and streptomycin administered within eight weeks. None of the eligible study participants was excluded.

Clinical validation was conducted on pre-treatment swab samples in PANTA (BD, Heidelberg, Germany) from 24 suspected BUD cases from Agogo Presbyterian Hospital (*n* = 14) and Tepa Government Hospital (*n* = 10), Ghana (Protocol S1). In addition, post-treatment swab samples from seven IS*2404* PCR confirmed BUD patients with incomplete wound healing were collected at week nine (Figures 1 and 2).

All clinical samples were subjected to routine diagnostics (microscopy and IS*2404* dry-reagent-based [DRB] PCR) at the Kumasi Centre for Collaborative Research (KCCR) [3].

### Primers and Probes

Primers and a hydrolysis probe (TibMolBiol, Berlin, Germany) for specific amplification of *M*. *ulcerans* 16S rRNA were designed using DNAsis Max (MiraiBio, San Francisco, USA) by alignment of 16S rRNA gene sequences (GenBank, National Center for Biotechnology Information [NCBI]) from closely related mycobacteria and other bacteria potentially contaminating the human skin (Table 2).

For simultaneous quantification by IS*2404* qPCR, the primers described by Fyfe et al. [9] were used in combination with a hydrolysis probe (Table 3) that was re-designed by DNAsis Max for thermodynamic reasons.

**Table 3:** Primers and probes.

Primer/Probe^a^	Sequence (5′–3′)	Target Gene^b^	Nucleotide Position^c^	Amplicon Size^d^
MU16S TFMU16S TRMU16S TP	CGA TCT GCC CTG CAC TTC CCA CAC CGC AAA AGC TT6 FAM-CAC AGG ACA TGA ATC CCG TGG TC-BBQ^e^	16S rRNA	4414800–44148174414718–44147344414740–4414762	100 bp
IS2404 TFIS2404 TRIS2404 TP2	AAA GCA CCA CGC AGC ATC T AGC GAC CCC AGT GGA TTG6 FAM-CCG TCC AAC GCG ATC GGC A-BBQ^e^	IS*2404*	96685–9666796627–9664496664–96646	59 bp
T13^f^T39^f^	TGC ACA CAG GCC ACA AGG GACG AAC GGG TGA GTA ACA CG	16S rRNA	4413906–44139254414822–4414840	935 bp

Table 3 indicates primers and probes designed for the 16S rRNA RT-qPCR, the primers described by Fyfe et al., and a re-designed hydrolysis probe used for the amplification, detection, and quantification of IS*2404* [9].

a TF, forward primer; TR, reverse primer; TP2, hydrolysis probe (TibMolBiol, Berlin, Germany).

b 16S rRNA, gene for the ribosomal 16S RNA detected as 16S cDNA; IS*2404*, insertion sequence *2404*.

c Nucleotide positions are provided for the first (IS*2404*) or single (16S rRNA) copy of the respective amplicon in *M. ulcerans* Agy99 (GenBank accession no. CP000325.1) as determined by BLASTN analysis within GenBank (NCBI) [13].

d bp, base pairs.

e 6 FAM, 6-Carboxyfluorescein fluorescent dye; BBQ, BlackBerry Quencher.

f Primers T13 (forward) and T39 (reverse) were used for the amplification of a 935-bp region of the *M. ulcerans* 16S rRNA gene, encompassing the region amplified by qPCR primers MU16S TF and MU16S TR, to generate single copy replicates. Furthermore, these primers were used for sequencing of the *M. ulcerans* 16S rRNA gene (Table 1).

### Combined RNA/DNA Extraction, Reverse Transcription, and Real-Time qPCR

Culture suspensions and swab samples were stabilized by RNA protect (Qiagen, Hilden, Germany) and subjected to AllPrep DNA/RNA extraction kit (Qiagen) (Protocols S1 and S2).

*M*. *ulcerans* whole transcriptome RNA from cultures and swab samples was transcribed to cDNA by QuantiTect Reverse Transcription Kit (Qiagen) including genomic DNA (gDNA) wipeout (Protocol S2). DNA and cDNA were subjected to IS*2404* qPCR and 16S rRNA RT-qPCR, respectively, with corresponding controls (Table 4, Protocols S3 and S4).

**Table 4:** Controls applied in 16S rRNA RT/IS*2404* qPCR.

Control	Purpose	Material	
		16S rRNA RT-qPCR^a^	IS*2404* qPCR^b^
gDNA wipeout control^c^	To exclude DNA contamination of RNA extracts	Aliquot of each RNA extract following gDNA wipeout before reverse transcription	NA
Internal positive control	To exclude false negative results due to inhibition	TaqMan exogenous internal positive control (IPC)^d^	TaqMan exogenous internal positive control (IPC)^d^
Positive run control	To ensure adequate performance of PCR	*M*. *ulcerans* cDNA^e^	Cloned IS*2404* standard
Negative no template control	To exclude contamination during PCR set up	H_2_O	H_2_O
Negative extraction control	To exclude contamination during extraction procedure	NA	Extract treated in the same way as samples

Table 4 indicates controls applied in 16S rRNA RT/IS*2404* qPCR. NA, not applicable.

a 16S rRNA RT PCR, reverse transcriptase real-time PCR targeting the 16S ribosomal RNA of *M. ulcerans*.

b IS*2404* qPCR, quantitative real-time PCR targeting the insertion sequence (IS) *2404* of *M. ulcerans*.

c gDNA, genomic DNA wipeout was conducted using DNAses provided in the QuantiTect Reverse Transcription Kit (Qiagen).

d TaqMan exogenous internal positive control (Applied Biosystems, Carlsbad, CA).

e cDNA, complementary DNA obtained through reverse transcription of *M. ulcerans* RNA by QuantiTect Reverse Transcription Kit (Qiagen).

### Intra- and Inter-Assay Variability

Intra- and inter-assay variability was assessed by testing of each sample in quadruplicate within one 96-well plate, repeated on three different days (Table 5).

**Table 5:** Intra- and inter-assay variability of the 16S rRNA RT/IS*2404* qPCR assay.

qPCR Target^a^	Standard No.	Run No. 1		Run No. 2		Run No. 3		Intra-Assay Variability		Inter-Assay Variability			
		Ct-range^b^	CV^c^	Ct-range^b^	CV^c^	Ct-range^b^	CV^c^	ΔCt max.^d^	CV max.^e^	Ct-range^f^	CV^g^	ΔCt max.^h^	CV max.^i^
16S rRNA	12345	0.230.090.120.150.07	0.500.190.180.220.10	0.120.160.060.170.15	0.480.300.200.250.20	0.170.190.200.120.16	0.420.350.320.220.20	0.23	0.49	0.550.240.310.750.71	1.330.530.551.150.92	0.75	1.33
IS*2404*	1234567	0.120.180.020.180.310.150.35	0.530.650.070.390.580.230.48	0.130.150.230.140.250.310.15	0.540.480.600.280.420.470.33	0.100.180.110.100.220.200.08	0.420.570.280.220.380.320.29	0.35	0.65	0.610.710.800.800.580.310.74	2.672.352.131.761.090.581.10	0.80	2.66

Table 5 shows intra- and inter-assay variability of the 16S rRNA RT/IS*2404* qPCR assay.

16S rRNA RT-qPCR: 16S rRNA gene standards (935 bp) were generated by conventional PCR according to Talaat et al. [12]. Quantification of PCR products was conducted by Picogreen fluorometry (Invitrogen) and copy numbers were calculated based on the known mass of one amplicon. Serial standards were prepared from PCR products in 5 Log dilutions ranging from 3E+6 (standard no. 1) to 3E+2 copies (standard no. 5) of the 16S rRNA amplicon (PCR template: 2 µl) and were subjected to the 16S rRNA RT-qPCR in quadruplicate on one 96-well plate to assess intra-assay variability. The runs were repeated on three days to determine the inter-assay variability between runs 1 through 3. The intra- and inter-assay variability of the 16S rRNA RT-qPCR was low with maximum coefficients of variation (CV) of 0.49 (intra-assay) and 1.33 (inter-assay).

IS*2404* qPCR: Cloned IS*2404* replicates (1,047 bp, complete sequence; *M. ulcerans* Agy99) were used as standards. Quantification of IS*2404* templates was conducted by Picogreen fluorometry (Invitrogen) and copy numbers were calculated based on the known mass of one template. Serial standards were prepared in 7 Log dilutions ranging from 2E+8 to 2E+2 copies of the IS*2404* (PCR template: 2 µl) and were subjected to the IS*2404* qPCR in quadruplicate on one 96-well plate to assess intra-assay variability. The runs were repeated on three days to determine the inter-assay variability between runs 1 through 3. The intra- and inter-assay variability of the IS*2404* qPCR was low with maximum CV of 0.65 (intra-assay) and 2.66 (inter-assay).

a 16S rRNA, target of the 16S rRNA RT-qPCR; IS*2404*, target of the IS*2404* qPCR.

b Ct-range, range of Ct-values of samples tested in the same dilution.

c CV, coefficient of variation of copy numbers from samples tested in quadruplicate of the same dilution.

d ΔCt max., maximum Ct-variation of all samples tested within one run.

e CV max., maximum CV of all samples tested within one run.

f Ct-range, range of Ct-values of samples tested in the same dilution within three runs.

g CV of samples in the same dilution tested within three runs.

h ΔCt max. of all samples tested within three runs.

i CV max. of all samples tested within three runs.

### Sensitivity

**Figure 3:**
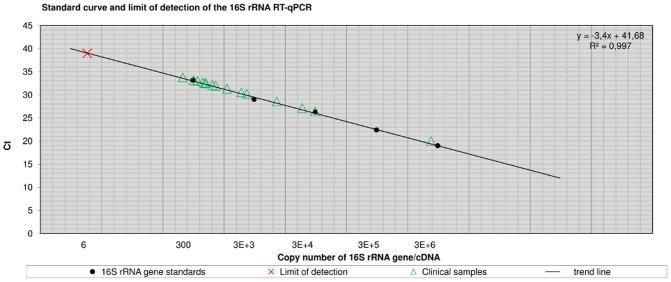
Standard curve and limit of detection of the 16S rRNA RT-qPCR. Figure 3 shows Ct-values of clinical samples plotted versus quantified 16S rRNA copy numbers. Standards for the 16S rRNA RT-qPCR were generated by conventional PCR amplification (Table 5). Log 10 fold serial dilutions (*n* = 5) were prepared ranging from 3E+6 to 300 copies of the 16S rRNA gene (PCR template: 2 µl) and were subjected to the assay in quadruplicate to generate a calibration curve. The regression line was y = −3.4x+41.68 with a coefficient of correlation >0.99 and the efficiency was E = 0.97. *M. ulcerans* whole genome extracts were quantified by means of IS*2404* qPCR and the analytical sensitivity was determined as limit of detection (LOD) by subjecting 10 aliquots of a dilution series containing 30, 15, 10, 8, 6, 3, or 2 copies of the 16S rRNA gene to the assay. The LOD was 6 copies of the target sequence. The copy number (*n* = 1) of the 16S rRNA gene per *M. ulcerans* genome was determined by copy number variation assay (unpublished data).

**Figure 4:**
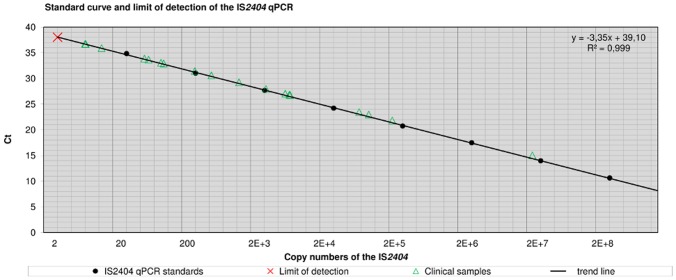
Standard curve and limit of detection of the IS*2404* qPCR. Figure 4 shows mean Ct-values of calibration standards and clinical samples plotted versus the quantified copy number of IS*2404*. Cloned IS*2404* templates were used as standards (Table 5). Log 10 fold serial dilutions (*n* = 8) were prepared ranging from 2E+8 to 20 copies of the IS*2404* (PCR template: 2 µl) and were subjected to the IS*2404* qPCR in quadruplicate to generate a calibration curve. The regression line was y = −3.35x+39.10 with a coefficient of correlation >0.99 and the efficiency was E = 0.97. The analytical sensitivity was determined as limit of detection (LOD) by subjecting 10 aliquots of a dilution series containing 10, 5, 4, 3, 2, or 1 copy of the IS*2404* to the assay. The LOD was 2 copies of the target sequence.

The analytical sensitivity was determined as lower limit of detection (LOD, lowest template concentration rendering amplification of 95% of samples) [10] for both qPCR components using 10-fold serial dilutions of cloned IS*2404* templates (GenExpress, Berlin, Germany) with known copy numbers (IS*2404* qPCR) and exactly quantified *M*. *ulcerans* whole genome DNA extracts from cultures (16S rRNA RT-qPCR). The LOD was two (IS*2404*) and six templates (16S rRNA gene), respectively (Figures 3 and 4).

*M*. *ulcerans* DNA and rRNA was detected in all culture extracts. Out of 24 pre-treatment swab samples, 18 (75.0%; 95%-CI: 57.7%–92.3%) had a positive IS*2404* qPCR result, 12 out of those were also positive in routine DRB PCR, and rRNA was detected in 15 out of these 18 samples (83.3%; 95%-CI: 66.1%–100%); quantification of the three negative samples revealed a bacillary load below the LOD of the 16S rRNA RT-qPCR (Table 6).

**Table 6:** Study participants, clinical information, and diagnostic results.

Clinical Data				Molecular Viability Assay^a^			Routine Diagnostics^b^	
No.^c^	BUD Patient^d^	Duration (Weeks)^e^	Category of Lesion^f^	IS*2404* [Ct]^g^	Bacillary Load^h^	16S rRNA^i^	MIC^k^	PCR^l^
1	No	NA	NA	Neg [NA]	NA	Neg	0	Neg
2	Yes	6	III	Pos [15,04]	>1000	Pos	+1	Pos
3	Yes	4	III	Pos [26,80]	584	Pos	+1	Pos
4	Yes	9	III	Pos [32,93]	6–10	Pos	0	Neg
5	Yes	4	I	Pos [35,94]	1–5	Neg	0	Neg
6	Yes	8	II	Pos [36,72]	1–5	Neg	0	Neg
7	Yes	2	I	Pos [36,74]	1–5	Neg	0	Neg
8	Yes	10	I	Pos [27,05]	497	Pos	+1	Pos
9	No	NA	NA	Neg [NA]	NA	Neg	0	Neg
10	Yes	3	I	Pos [30,61]	42	Pos	+1	Pos
11	Yes	8	II	Pos [33,89]	6–10	Pos	0	Neg
12	Yes	9	I	Pos [33,68]	6–10	Pos	0	Neg
13	Yes	3	III	Pos [29,27]	106	Pos	+1	Pos
14	Yes	3	I	Pos [27,98]	261	Pos	+1	Pos
15	Yes	1	I	Pos [26,85]	571	Pos	+1	Pos
16	Yes	2	I	Pos [33,07]	6–10	Pos	0	Pos
17	Yes	2	II	Pos [31,44]	24	Pos	+1	Pos
18	Yes	3	II	Pos [21,85]	>1000	Pos	+2	Pos
19	Yes	4	III	Pos [22,98]	>1000	Pos	+1	Pos
20	Yes	3	I	Pos [23,47]	>1000	Pos	+2	Pos
21	No	NA	NA	Neg [NA]	NA	Neg	0	Neg
22	No	NA	NA	Neg [NA]	NA	Neg	0	Neg
23	No	NA	NA	Neg [NA]	NA	Neg	0	Neg
24	No	NA	NA	Neg [NA]	NA	Neg	0	Neg

Table 6 shows suspected BUD cases with ulcerative lesions enrolled in the pre-treatment cohort (Figure 1), clinical information, and diagnostic results. Swab samples from 24 suspected BUD cases were subjected to 16S rRNA RT/IS*2404* qPCR viability assay (swab 1 in PANTA), microscopic examination and enumeration of acid fast bacilli (AFB) following Ziehl-Neelsen staining (swab 2, direct smear), and conventional IS*2404* dry-reagent-based (DRB) PCR (swab 3 in Cell Lysis Solution [Qiagen]). 18 patients were laboratory confirmed by IS*2404* qPCR and 15 out of those were RNA positive; the quantification by IS*2404* qPCR revealed a bacillary load (1–2 bacilli per sample) below the lower limit of detection of the RNA assay for samples from three RNA negative patients. All samples from six IS*2404* qPCR negative study participants were also RNA negative. Direct correlation of AFB enumeration with IS*2404* qPCR quantification is not feasible due to inhomogeneous distribution of *M. ulcerans* in different clinical samples. NA, not applicable; Neg, negative test result; Pos, positive test result.

a Results of the 16S rRNA RT/IS*2404* qPCR viability assay. Clinical swab samples in PANTA were directly processed at KCCR, and *M. ulcerans* DNA and cDNA were transported to DITM and subjected to qPCR.

b Routine diagnostics were conducted following standardized procedures at KCCR [3].

c No., consecutive number of study participants.

d Yes, IS*2404* qPCR confirmed BUD patients; No, IS*2404* negative study participants.

e Duration of disease before presentation of study participants in weeks.

f Category of lesion according to the World Health Organization's clinical criteria [1].

g Results of the IS*2404* qPCR with corresponding cycle threshold (Ct)-values.

h The bacillary load in the respective swab samples (No. 2) was estimated on the basis of IS*2404* quantification given an IS*2404* copy number of 209 copies per *M. ulcerans* genome [9]. For bacterial numbers <10 ranges were estimated.

i Results of the 16S rRNA RT-qPCR.

k MIC, microscopic detection and enumeration of AFB was conducted at KCCR including external quality assurance by DITM. The following scale was applied: 0 = negative, +1 = 10–99 AFB/100 fields, +2 = 1–10 AFB/1 field, +3 = more than 10 AFB/1 field.

l PCR, conventional, single target gel-based IS*2404* DRB PCR.

All seven post-treatment swab samples were IS*2404* qPCR positive and 16S rRNA negative.

### Specificity

Analysis of DNA extracts revealed 100% specificity for the combined assay. *M*. *marinum* (human isolate) was amplified by 16S rRNA RT-qPCR; however, simultaneous IS*2404* qPCR was negative (Table 2).

### Bacillary Survival Times

To investigate the effect of sample transport on bacillary survival, mycobacteriological transport media (PANTA and LTM) [3] were spiked with viable *M. ulcerans* and stored at 4°C and 31°C. RNA was detectable in both media for >4 weeks (4°C and 31°C).

After heat-inactivation of *M. ulcerans–*spiked PANTA-samples, RNA positivity decreased significantly within 12 h, whereas DNA was still detectable after seven days.

## Future Application

The assay will support clinicians in classification of secondary lesions and selection of adequate clinical management strategies and provides a powerful tool for clinical research evaluating novel treatment regimens (Box 1).

**Box 1. Advantages and Disadvantages of the Molecular Viability Assay**

*Advantages*

- Provides a rapid, sensitive, and specific tool to detect viable bacilli in clinical samples of BUD patients, thus offering an alternative to cultures.
- Supports classification of secondary BUD lesions and monitoring of treatment success.

*Disadvantages*

- Current test format requires well equipped laboratory with real-time PCR facilities.
- Costs per test (approximately 14 €) may limit the applicability.

Through analysis of sequential samples collected during antimycobacterial treatment, the assay will be employed to determine the proportional decrease of bacterial viability over time and to establish laboratory-based evidence for optimal time-points to collect follow-up samples for treatment monitoring.

Whereas the current format of the assay is restricted to reference laboratories, sample collection on FTA cards in combination with isothermal dry-reagent-based reverse transcription and amplification formats would facilitate processing of samples also at a peripheral level and at lower costs.

## Conclusions

The novel combined 16S rRNA RT/IS*2404* qPCR assay proved to be highly sensitive, specific, and efficient in detecting viable *M. ulcerans* in clinical samples under field conditions. The assay is applicable for classification of secondary lesions and monitoring of treatment success and provides a powerful tool for clinical research.

### GenBank Accession Numbers

Genes or DNA sequences of mycobacterial strains used in this study were retrieved from GenBank (NCBI) [13]. The respective sequences and accession numbers are summarized in Table S1.

## Supporting Information

Protocol S1 Preparation of PANTA transport medium and stabilisation of *M*. *ulcerans* RNA/DNA in swab samples and culture suspensions.
(PDF)

Protocol S2 Simultaneous RNA/DNA extraction from swab samples and reverse transcription of whole transcriptome RNA from *M. ulcerans*.
(PDF)

Protocol S3 Combined 16S rRNA RT/IS*2404* qPCR assay.
(PDF)

Protocol S4 16S rRNA RT/IS*2404* qPCR run protocol.
(XLS)

Table S1 GenBank accession numbers.
(DOC)
