# Supplementary material for: Detection of Viable Mycobacterium ulcerans in Clinical Samples by a Novel Combined 16S rRNA Reverse Transcriptase/IS2404 Real-Time qPCR Assay
Source: PLoS Negl Trop Dis. 2012 Aug 28;6(8):e1756. doi: 10.1371/journal.pntd.0001756 (PMC3429398; doi:10.1371/journal.pntd.0001756)
Supplement: Protocol S1 — Preparation of PANTA transport medium and stabilisation of M. ulcerans RNA/DNA in swab samples and culture suspensions. (PDF) [file pntd.0001756.s001.pdf]

Protocol S1

Preparation of PANTA transport medium and stabilisation of *M. ulcerans* RNA/DNA in swab samples and culture suspensions

DITM (LMU), KATH and KCCR (KNUST) **v1 03.01.2012**

1. General considerations

This document describes the standard operating procedure for the preparation of PANTA transport medium for viable *M. ulcerans* in clinical samples and stabilisation of swab samples and culture suspensions for combined mycobacterial RNA/DNA extraction in two steps:

1) Preparation of PANTA transport medium for swab samples

2) Stabilisation of *M. ulcerans* from culture or swab samples for RNA/DNA extraction

▲ = ***Note !***

2. Reagents, material and instruments

2.1 PANTA transport medium

- Dubos Broth Base, 500g (BD, Heidelberg, Germany, ref# 238510)
- Dubos Medium Albumin, 12x20 ml (BD, ref# 230910)
- BBL MGIT PANTA, 6 pcs. (BD, ref# 245114)
- Glycerol (1,2,3-Propantriol), 99.5% p.a., 1L (e.g. Roth, Karlsruhe, Germany, ref# 3783.1)
- H2O, 1L
- 2 ml certified RNAse/DNAse free screw cap tube (Sarstedt, Nümbrecht, Germany, ref# 72.694.100)
- Rack for tubes, sterile 1L sterile glass bottle, Laminar Flow cabinet

2.2 Stabilisation of swab samples for RNA/DNA extraction

- Medical swab, 165 mm, cotton/wood, sterile (Milan, Basel, Switzerland, ref# EBC-150)
- 2 ml tubes with 500 µl PANTA (alternatively LTM)
- RNA Protect Bacteria reagent, 2x100ml (Qiagen, Hilden, Germany, ref# 76506)
- Biosafety cabinet class II
- Centrifuge
- Thermomixer
- Freezer (-20°C/-70°C)

3. Preparation of PANTA transport medium

▲ ***Work under sterile conditions in a laminar Flow!***

1) Distil 850 ml water and filter through sterile bottle top filter.
2) Filter glycerol through sterile bottle top filter (this will take a while due to the consistency).

▲ ***Glycerol is heat labile!***

3) Dissolve 1.5 g Dubos Broth Base in 204 ml distilled water and autoclave for 15 min. at 121°C. Allow the solution to cool down to approx. 50°C before adding 12 ml of filtered glycerol and 24 ml Dubos Medium Albumin.
4) Dissolve 2 vials of BBL MGIT PANTA (Polymyxin B, Amphotericin B, Nalidixic acid, Trimethoprim, Azlocillin) in 3 ml filtered distilled water each to obtain PANTA antibiotic mixture.
5) For the preparation of PANTA transport medium add 6 ml of PANTA antibiotic mixture to the autoclaved Dubos Broth Base/glycerol mixture under sterile conditions.
6) To check sterility add 1 ml PANTA transport medium under sterile conditions to two 2 ml screw cap tubes each and incubate at 36°C for 24h (tube 1) and 1 week (tube 2) respectively. A clear medium indicates sterility while a turbid medium indicates contamination with bacteria (tube 1) and/or fungi

(tube 2). In case of contamination discard the medium and prepare a new batch PANTA transport medium before use.

Store the medium at 4-8°C for max. 3 months in a sterile glass bottle labelled “PANTA transport medium” with the date of preparation. Aliquot 500 µl PANTA transport medium in 2 ml screw cap tubes labelled “PANTA” under sterile conditions.

▲*Alternatively Liquid Transport Medium (LTM)*[1] *can be used if routinely applied for cultures in the respective laboratory*.

4. Stabilisation of swab samples for RNA/DNA extraction

4.1.a. Stabilization of swab samples

1a) Collect swab(s) from BUD suspected lesions by circling the entire undermined edge of the ulcer according to routine procedure.
2a) Store swab stick in 2 ml tubes containing 500µl PANTA transport medium (alternatively 500µl LTM)

▲*The swab can be transported at ambient temperature and may be stored for up to 4 weeks at 4°C, however it is recommended to* ***apply the following steps directly upon arrival*** *of clinical samples at the laboratory!*

▲***Work in a biosafety cabinet class II***

3a) Add 1 ml RNA Protect Bacteria reagent to each swab sample in 500 µl PANTA transport medium (2:1), vortex vigorously for 10 sec.
4a) Incubate specimens for 5 min. at ambient temperature, vortex every minute for 10 sec.
5a) Inactivate samples at 95°C for 10 min., incubate on ice for 5 min.
6a) Pellet material by centrifugation at 5000g at ambient temperature for 5 min., remove swab stick carefully and discard supernatant, keep samples on ice and continue RNA/DNA extraction with pellet (see protocol S2) or freeze pellets at -20°C for up to 1 month (alternatively at -70°C for up to 12 months).

4.1.b. Stabilization of culture suspension

1b) Add 1 ml RNA Protect Bacteria reagent to tubes containing 500 µl PANTA transport medium.

▲***Work in a biosafety cabinet class II***

2b) Suspend one loop-full of *M. ulcerans* culture material in the mixture from step 1).
3b) Vortex vigorously for 10 sec.
4b) Incubate suspension at ambient temperature for 5 min., vortex every minute for 10 sec.
5b) Inactivate suspensions at 95°C for 5 min., incubate on ice for 5 min.
6b) Pellet material by centrifugation at 5000g at ambient temperature for 5 min., discard supernatant, keep samples on ice and continue RNA/DNA extraction with pellet (see protocol S2) or freeze pellets at -20°C for up to 1 month (alternatively at -70°C for up to 12 months).

[1]Fine-Needle Aspiration, an Efficient Sampling Technique for Bacteriological Diagnosis of Nonulcerative Buruli Ulcer (2009) Eddyani M, Fraga AG, Schmitt F, Uwizeye C, et al. J Clin Microbiol. 2009 June; 47(6): 1700–1704.
