# Supplementary material for: Detection of Viable Mycobacterium ulcerans in Clinical Samples by a Novel Combined 16S rRNA Reverse Transcriptase/IS2404 Real-Time qPCR Assay
Source: PLoS Negl Trop Dis. 2012 Aug 28;6(8):e1756. doi: 10.1371/journal.pntd.0001756 (PMC3429398; doi:10.1371/journal.pntd.0001756)
Supplement: Protocol S2 — Simultaneous RNA/DNA extraction from swab samples and reverse transcription of whole transcriptome RNA from M. ulcerans. (PDF) [file pntd.0001756.s002.pdf]

Simultaneous RNA/DNA extraction from swab samples and reverse transcription of whole transcriptome RNA from *M. ulcerans*

DITM (LMU), KATH and KCCR (KNUST) **v1 03.01.2012**

1. General considerations

This document describes the standard operating procedure for simultaneous extraction of whole genome DNA and whole transcriptome RNA (incl. reverse transcription to cDNA) of *M. ulcerans* from clinical swab samples in two steps:

1) Simultaneous RNA/DNA extraction from *M. ulcerans*

2) Reverse transcription of *M. ulcerans* whole transcriptome RNA to cDNA

Follow all general precautions for RNA works and do not process more than 6-8 samples within one extraction procedure to adhere to quick processing of samples!

▲ = ***Note !***

2. Material, reagents and instruments

2.1 Simultaneous RNA/DNA extraction from M. ulcerans

- AllPrep DNA/RNA Micro Kit (Qiagen, Hilden, Germany, ref# 80284)
- Cell lysis solution (CLS), 125 ml (Qiagen, ref# 158906)
- Proteinase K (>600 mAU/ml, solution), 2 ml (Qiagen, ref# 19131)
- Lysozyme, 1g (AppliChem, Darmstadt, Germany, ref# A4972,0001)
- Beta-(2)-Mercaptoethanol, 25 ml (AppliChem, ref# A1108,0025)
- Ethanol 96%, p.a., 500 ml (AppliChem, ref# A1615,0500GL)
- RNase Away, 2.5 L (Roth, Darmstadt, Germany, ref# A998.1)
- DEPC (Diethylpyrocarbonate) treated water, 1L (Roth, ref# T143.3)
- Reaction tubes, 1.5 ml, certified DNase/RNase free (Kisker, Steinfurt, Germany)
- 10 µl, 100 µl, 1000µl pipette filter tips, certified DNase/RNase free (Kisker)
- Syringes, sterile, 0.5 ml (Braun, Melsungen Germany)
- 21 Gauge needles, sterile (Braun)
- Sharps container
- Disposable gloves, nitrile, non-sterile
- Small ice container, crushed ice
- Tube rack, centrifuge, thermomixer, Laminar Flow, Fume hood, Vortex

2.2 Reverse transcription of M. ulcerans whole transcriptome RNA to cDNA

- QuantiTect Reverse Transcription Kit (Qiagen, ref# 205311)
- Reaction tubes, 0.2 ml, certified DNase/RNase free (Kisker, ref# GS001F)
- Reaction tubes, 1.5 ml, certified DNase/RNase free (Kisker)
- 10 µl pipette filter tips, certified DNase/RNase free (Kisker)
- 0.5-10 µl Pipette
- Thermomixers
- Small ice container, crushed ice

3. Simultaneous RNA/DNA extraction from *M. ulcerans*

3.1. Preparations

▲*All steps are conducted at **ambient temperature (AT)** if not explicitly indicated otherwise. Work quickly, but carefully! Perform centrifugation at 20 - 25° C, do not cool centrifuge below 20°C! Follow all general precautions for working with RNA! Wear gloves at all times and change them frequently! Clean the working space with RNAse Away before getting started.*

<u>The AllPrep DNA/RNA Micro Kit contains:</u>

Buffers: RLT Plus, RPE, RW1, AW1, AW2, EB and RNAse free water

DNA and RNeasy spin columns, collection tubes and 1.5 ml reaction tubes

<u>Before using the AllPrep DNA/RNA Micro Kit for the first time</u>:

I) Add ethanol 96%: 44 ml to Buffer RPE, 25 ml to Buffer AW1 and 30 ml to Buffer AW2.

II) Prepare 30 ml ethanol 80% by adding 5 ml DEPC treated water to 25 ml ethanol 96%.

III) Prepare 48 ml ethanol 70% by adding 13 ml DEPC treated water to 35 ml ethanol 96%.

IV) Generate 15 mg/ml CLS+L (CLS with lysozyme) buffer by adding 15mg lysozyme per ml CLS buffer, store at -20°C for up to 6 months.

<u>General preparation prior to each extraction:</u>

A) ▲ ***Use a fume hood and wear nitrile gloves!***

Add **3.5µl x n** (n = no. of samples processed) **ß-Mercaptoethanol** (ß-ME) to **350µl x n Buffer RLT Plus**. Buffer RLT Plus containing ß-ME is stable at room temperature for up to one month.

B) Clean all work surfaces with RNase Away reagent.

C) To prepare RNase-free syringes and needles pass DEPC-treated water through entire syringe and discard DEPC-treated water.

D) Label DNA spin columns & collection tubes.

E) Label RNeasy spin columns.

F) Prepare crushed ice in a reaction tube container.

3.2 Lysis

1) If applicable thaw frozen pellets on ice.
2) Add 100µl CLS+L Buffer and 20µl Proteinase K to each pellet on ice, vortex for 15 sec.
3) Incubate 5 min at AT, vortex 15 sec every minute.
4) Incubate 5 min at 45°C in thermomixer at high speed.
5) Add 350µl Buffer RLT Plus (containing ß-ME) under fume hood, vortex vigorously for 15 sec.
6) Homogenise lysate by slowly (to avoid foaming!) passing it 5 times through the DEPC-treated 0.9 mm needle with syringe.
7) Transfer homogenized lysate from the syringe directly into labelled AllPrep DNA spin columns in a labelled 2 ml collection tube.
8) Centrifuge at 9000 g for 30 sec at AT, check if all liquid has passed through membrane, if not: repeat this step.
9) Transfer flow-through in collection tubes immediately on ice for subsequent RNA purification (3.3). Place AllPrep DNA spin columns in new collection tubes, store at 4 – 8°C (for up to 24h) for subsequent DNA purification (5.2).

3.3 RNA extraction and purification

▲ ***All centrifugations are conducted at ambient temperature (AT)!***

10) Add 350 µl ethanol 70% to the flow-through in collection tubes from step 9, pipet 5 times up and down to mix.
11) Pipet samples quickly - but carefully - to labelled RNeasy spin columns. Avoid touching the membrane with the filter tip.
12) Centrifuge at 9000g for 15 sec, discard flow-through and transfer RNeasy spin columns to new collection tubes.
13) Add 700 µl Buffer RW1 to the RNeasy spin columns.
14) Centrifuge at 9000g for 15sec, discard flow-through and transfer RNeasy spin columns to new collection tubes.
15) Add 500 µl Buffer RPE to RNeasy spin columns.
16) Centrifuge at 9000g for 15sec, discard flow-through and transfer RNeasy spin columns to new collection tubes.
17) Add 500 µl ethanol 80% to RNeasy spin columns.
18) Centrifuge at 9000g for 2 min, discard flow-through with collection tubes.
19) Place spin columns in new collection tubes, open lid and centrifuge at 9000g for 5 min.
20) Discard collection tubes with flow-through.
21) Place spin columns in labelled 1.5ml Eppendorf tubes (provided with the kit), pipet 25 µl RNase-free water directly onto the spin column membrane. Avoid touching the membrane with the filter tip!
22) Centrifuge at 9000g for 1 min, to elute RNA. Place eluates immediately on ice!
23) Repeat step 21) for a second elution with 25 µl RNase-free water. Place eluates immediately on ice or freeze at -70°C.

4. Reverse transcription of *M. ulcerans* whole transcriptome RNA to cDNA

4.1. Kit

QuantiTect Reverse Transcription Kit contains: RT Primer Mix, RT Buffer, RNAse free water, gDNA Wipeout Buffer

4.2. Preparations

I) Kit: Premix RT Primer Mix and RT Buffer in a 1:4 ratio (n x 1 µl RT Primer Mix plus 4 x n x 1 µl RT Buffer; n= No. of reactions) and label with RT reaction buffer (stable at -20°C for 12 months).

II) In case the thermomixer does not provide an adapter for 0.2 ml tubes: Prepare 1.5 ml reaction tubes filled with RNAse free water (DEPC treated water) according to the sample size and incubate in thermomixer(s) at 42°C (and 95°C).

III) Label 0.2 ml PCR reaction tubes.

4.3. Reverse transcription

▲ ***Work on ice!***

Genomic DNA wipeout (gDNA wipe out):

24) Add 2 µl gDNA Wipeout Buffer to 12 µl template RNA from step 23 in labelled 0.2 ml reaction tubes (mix by flicking tubes, centrifuge briefly to ensure that all potentially contaminating DNA is in the reaction mix).
25) Store remaining RNA on ice for subsequent, optional quantification or gel-electrophoresis for quality control purposes or freeze at -70°C.
26) Incubate at 42°C for 5 min.
27) Inactivate reactions at 95°C for 3 min and put samples immediately on ice.

28) Aliquot 1 wipeout control (2 µl) per sample (wipeout control = genomic DNA wipeout without subsequent reverse transcription) as negative control and store at -20°C.

Reverse transcription:

29) Add 1 µl Reverse Transcriptase (keep RT always on ice!), 5 µl premixed RT reaction buffer and 2 µl RNAse free water to the samples from step 28. Incubate at 42°C for 15 min.
30) Inactivate Reverse Transcriptase at 95°C for 3 min and store transcribed cDNA on ice for subsequent 16S rRNA RT-qPCR or at -20°C for long-term storage.

▲ *Optionally conduct RNA quality control procedures: Quantification of RNA by fluorometry (e.g. Qubit, Invitrogen) and/or RNA gel-electrophoresis.*

5. DNA extraction

▲ *Reagents and material as described under 2.1 & 2.2. Samples from step 9 (3.2) are processed!*

5.1 Preparations

I) Preheat Buffer EB to 70°C (50 µl + 10 % per sample) prior to DNA elution.

5.2 DNA extraction and purification

31) Add 500µl Buffer AW1 to labelled AllPrep DNA spin columns from step 9 (3.2).
32) Centrifuge at 9000 g for 15sec, discard flow-through.
33) Add 500µl Buffer AW2 to spin columns.
34) Centrifuge at 9000 g for 2 min, discard collection tube with flow-through. (If column contacts flow-through: empty the collection tube and centrifuge the spin column again at 9000 g for 1 min).
35) Place spin columns in labelled 1.5 ml reaction tubes and add 50µl Buffer EB (preheated to 70°C, see 5.1, I) directly onto spin column membrane.
36) Incubate 2 min at AT.
37) Centrifuge at 9000 g for 1 min.
38) Discard AllPrep DNA spin column, DNA is in eluate.
39) Store at 4 - 8°C for subsequent IS*2404* qPCR and/or at -20°C for long-term storage.
