# Supplementary material for: Detection of Viable Mycobacterium ulcerans in Clinical Samples by a Novel Combined 16S rRNA Reverse Transcriptase/IS2404 Real-Time qPCR Assay
Source: PLoS Negl Trop Dis. 2012 Aug 28;6(8):e1756. doi: 10.1371/journal.pntd.0001756 (PMC3429398; doi:10.1371/journal.pntd.0001756)
Supplement: Protocol S3 — Combined 16S rRNA RT/IS2404 qPCR assay. (PDF) [file pntd.0001756.s003.pdf]

LMU / BuruliVac	**Protocol S3**	KCCR
	**Combined 16S rRNA RT/IS*2404* qPCR assay**	
**DITM (LMU), KATH and KCCR (KNUST)**		**v1 03.01.2012**

1. General considerations

This document describes the standard operating procedure for the application of the combined 16S rRNA RT/IS*2404* qPCR assay on a Bio-Rad CFX96 real-time PCR detection system. The assay detects *M. ulcerans* cDNA (16S rRNA) and DNA (IS*2404*) from the same clinical sample or culture suspension. The assay consists of two amplification procedures:

1) 16S rRNA RT-qPCR

2) IS*2404* qPCR

The "PCR Master Mix" must be prepared in a laboratory free of DNA amplicons.
The laboratory must be equipped with lab coats, gloves and all necessary laboratory items exclusively used in this room.

▲ = ***Note !***

2. Preparations

2.1 Reagents & Material

- Primer IS2404 TF[1]: 5' aaa gca cca cgc agc atc t'3 (TibMolBiol, Berlin, Germany)
- Primer IS2404 TR[1]: 5' agc gac ccc agt gga ttg'3 (TibMolBiol)
- Probe IS2404 TP2: 5' FAM-ccg tcc aac gcg atc ggc a-BBQ'3 (TibMolBiol)
- Primer MU16 TF: 5' cga tct gcc ctg cac ttc'3 (TibMolBiol)
- Primer MU16 TR: 5' cca cac cgc aaa agc tt'3 (TibMolBiol)
- Probe MU16 TP: 5' 6FAM-cac agg aca tga atc ccg tgg tc-BBQ'3 (TibMolBiol)
- 5x HOT FIREPol® Probe qPCR Mix Plus (no ROX), 1 ml (SolisBioDyne, Tartu, Estonia, ref# 08- 15-00001)
- Exogenous internal positive control (IPC) reagent (VIC), 200 rct. (Invitrogen, Karlsruhe, Germany,
  ref# 4308323)
- ***Optional*** *for exact quantification*: IS*2404* Standard (GenExpress, Berlin, Germany)
- DEPC (Diethylpyrocarbonate) treated water, 1 L (Carl Roth, Essen, Germany, ref# T143.3)
- Reaction tubes, 1.5 ml, DNase/RNase free (Kisker, Steinfurt, Germany)
- 96 well multiplates, clear, with seals (BioRad, ref# MLL9601)
- 10 µl, 100 µl, 1000µl pipettes
- 10 µl, 100 µl, 1000µl pipette filter tips, DNase/RNase free (Kisker)
- Disposable gloves, non-sterile
- BioRad CFX96 real-time PCR detection system, centrifuge for multiplates, mini table centrifuge, Vortex, tube rack

<u>5x HOT FIREPol® Probe qPCR Mix Plus contains:</u>

- HOT FIREPol® DNA Polymerase
- 5 x Probe qPCR buffer
- 15 mM $MgCl^2$, 1 x PCR solution – 3 mM $MgCl^2$
- dNTPs, including dTTP which improves reaction sensitivity and efficiency

<u>Exogenous internal positive control (IPC) reagent contains:</u>

Exo IPC Mix with a 5'VIC-3'TAMRA labelled probe incl. corresponding primers and Exo IPC DNA.

2.2 Storage and preparation of reagents

Primers, probes, qPCR Mix Plus and Exo IPC Mix must be stored in aliquots at -20 °C in a laboratory free of DNA amplicons; store primers and probes in the dark. The EXO IPC DNA is stored in the DNA extraction room at -20°C.

3. Setting up the qPCR reactions

▲ ***The following steps are performed in the "Master Mix laboratory"!***

The reagents are thawed and a master mix of all reagents is prepared. Therefore the following protocols have to be multiplied by the number of samples to be tested (incl. all controls plus 10%).

A TaqMan exogenous internal positive control (IPC) is used in each amplification reaction to exclude false negative results due to inhibition (optionally the Exo IPC DNA can be already added prior to extraction to control the performance of the extraction procedure). Cloned IS*2404* standards and *M. ulcerans* whole transcriptome cDNA are used as positive run controls for the IS*2404* and 16S rRNA qPCR assay, respectively. To exclude contaminations with *M. ulcerans* DNA no template controls (containing H_2O instead of template; one control for each assay) and negative extraction controls (IS*2404* qPCR) are processed in the same way like the samples. To exclude DNA contamination of RNA extracts one negative wipeout control (i.e. aliquot of each extract following gDNA wipeout before reverse transcription) per cDNA sample is subjected to IS*2404* qPCR.

Before adding templates to the respective reaction mix, a PCR protocol indicating sample IDs and numbers of wells is prepared (e.g. S4 - 16S rRNA RT/IS*2404* qPCR run protocol). If using protocol S4, master mix calculation follows automatically after sample ID's are entered.

To avoid any contamination, first cDNA samples followed by negative and wipeout controls are added to the master mixes and the respective wells are sealed before DNA extracts and positive controls are added.

Master Mix 16S rRNA RT qPCR

**Reagent**	**per reaction in µl**	**Stock solution**	**Final conc.**
DEPC water	8,60	-	-
MU16S TF	1,00	10µM	0,5µM
MU16S TR	1,00	10µM	0,5µM
MU16S TP	1,00	5µM	0.25µM
qPCR Mix Plus	4,00	5 U/µl	0,025 U/µl
Exo IPC Mix	2,00	10x	1x
Exo IPC DNA	0,40	50x	1x
**Total vol. Mastermix [µl]**	**18,00**		
**Template cDNA**	**2,00**		
**Final volume**	**20,00**		

Master Mix IS*2404* qPCR

**Reagent**	**per reaction in µl**	**Stock solution**	**Final conc.**
DEPC water	8,60	-	-
IS2404 TF	1,00	10µM	0,5µM
IS2404 TR	1,00	10µM	0,5µM
IS2404 TP2	1,00	5µM	0.25µM
qPCR Mix Plus	4,00	5 U/µl	0,025 U/µl
Exo IPC Mix	2,00	10x	1x
Exo IPC DNA	0,40	50x	1x
**Total vol. Mastermix [µl]**	**18,00**		
**Template DNA**	**2,00**		
**Final volume**	**20,00**		

The Master Mix is prepared in a 1.5 ml reaction tube for each assay. Exo IPC DNA is added to the Master Mix in the DNA extraction laboratory. The 96 well multiplates are filled with 18µl of the reaction mix according to the sample size (including all controls). Following addition of templates the 96 well multiplate is sealed (wear gloves!) and centrifuged at 5000 x g for 2 min.

The **BioRad CFX96** (alternatively a comparable real-time detection system) is programmed according to the manufacturer's specifications using the following settings:

**Steps**	**Temperature**	**Duration**	**Number of cycles**
**1. Initial Denaturation**	95°C	15 minutes	1
**2. Denaturation**	95°C	15 seconds	40
**3. Annealing & Extension**	60°C	60 seconds	
**Activate detection of channels VIC and FAM for all wells following each cycle after step 3**			

The 96-well multiplate is transferred to the BioRad CFX96 and the programme is launched.

4. Interpretation of results

An exponential curve indicates a positive signal from specific amplification. A negative result produces no curve but a detection line below the threshold.

Table 1 and 2 provide an overview of all possible interpretations of IS*2404* qPCR and 16S rRNA RT qPCR results respectively.

Table 1. Interpretation of IS*2404* qPCR results

**Type of sample**	**FAM signal**	**VIC signal**	**Interpretation**
M. ulcerans DNA from clinical samples or cultures; positive, wipeout or negative controls*	positive	positive	Positive qPCR reaction
	positive	negative	Positive qPCR reaction
	negative	positive	Negative qPCR reaction
	negative	negative	Reaction inhibited**

*The positive run controls must result in a positive PCR reaction. The negative controls (i.e. no template control, negative extraction control and wipeout control) must remain negative. A positive "no template control" indicates contamination of reagents, a positive extraction control indicates cross-contamination during the extraction procedure and a positive wipeout control indicates DNA contamination of RNA extracts.
**Inhibited reactions must be repeated in a tenfold dilution.

Table 2. Interpretation of 16S rRNA RT-qPCR results

Type of sample*	FAM signal	VIC signal	Wipe out control*	Interpretation***
M. ulcerans cDNA from clinical samples or cultures	positive	positive	negative	Positive PCR reaction
	positive	negative	negative	Positive PCR reaction
	negative	positive	negative	Negative PCR reaction
	negative	negative	negative	Reaction inhibited
	positive	positive	positive	DNA contamination
	positive	negative	positive	DNA contamination
	negative	positive	positive	DNA contamination
	negative	negative	positive	Reaction inhibited and DNA contamination
Positive run control and negative no template control	positive	positive	NA	Positive PCR reaction
	positive	negative	NA	Positive PCR reaction
	negative	positive	NA	Negative PCR reaction
	negative	negative	NA	Reaction inhibited

*The positive run controls must result in a positive PCR reaction. The negative controls (i.e. no template control) must remain negative. A positive “no template control” indicates DNA contamination of reagents.
**IS*2404* qPCR result of corresponding gDNA Wipe out controls.
***Inhibited reactions (usually not seen with cDNA) must be repeated in a tenfold dilution.
NA, not applicable

5. References

1. Fyfe JA, Lavender CJ, Johnson PD, Globan M, Sievers A, et al. (2007) Development and application of two multiplex real-time PCR assays for the detection of Mycobacterium ulcerans in clinical and environmental samples. Appl Environ Microbiol. 73(15):4733-40.
