# Supplementary material for: Detection of Viable Mycobacterium ulcerans in Clinical Samples by a Novel Combined 16S rRNA Reverse Transcriptase/IS2404 Real-Time qPCR Assay
Source: PLoS Negl Trop Dis. 2012 Aug 28;6(8):e1756. doi: 10.1371/journal.pntd.0001756 (PMC3429398; doi:10.1371/journal.pntd.0001756)
Supplement: Table S1 — GenBank accession numbers. (DOC) [file pntd.0001756.s005.doc]

**Table S1. GenBank accession numbers.**

| **Species** | **Strain** | **Accession No.^a^** |
| --- | --- | --- |
| *M. abscessus* | PCH-043 | JN400392.1 |
| *M. africanum* | GM041182 | FR878060.1 |
| *M. avium* | Myc 373 | AF410479.1 |
| *M. bovis* | NA | M20940.1 |
| *M. chelonae* | PCH-036 | JN400395.1 |
| *M. fortuitum* | FMUNAM27 | JN049501.1 |
| *M. gordonae* | FMUNAM48 | JN049504.1 |
| *M. kansasii* | DSM44162 | NR_042164.1 |
| *M. leprae* | NA | AL450380.1 |
| *M. liflandii* | NA | AY500838.1 |
| *M. malmoense* | NA | AF152560.1 |
| *M. marinum* | DL045 | AF456241.1 |
| *M. marinum* | M | CP000854.1 |
| *M. microti* | ATCC 19422 | NR_025234.1 |
| *M. pseudoshottsii* | L15 | NR_042988.1 |
| *M. scrofulaceum* | ATCC 19981 | GQ153271.1 |
| *M. smegmatis* | FMUNAM18 | JN049499.1 |
| *M. szulgai* | ATCC 35799 | NR_026080.1 |
| *M. tuberculosis* | NA | CP002992.1 |
| *M. ulcerans* | Agy99 | CP000325.1 |
| *M. xenopi* | DSM43995 | NR_042163 |

Table S5 indicates GenBank (National Center for Biotechnology Information) accession numbers for genes and DNA sequences and corresponding mycobacterial species used in this study. *M.*, mycobacterium; NA, not applicable.

^a^GenBank accession numbers [13].
